# Structural insights into human MHC-II association with invariant chain

**DOI:** 10.1073/pnas.2403031121

**Published:** 2024-04-30

**Authors:** Nan Wang, Deepa Waghray, Nathanael A. Caveney, Kevin M. Jude, K. Christopher Garcia

**Affiliations:** ^a^Department of Molecular and Cellular Physiology, Stanford University School of Medicine, Stanford, CA 94305; ^b^HHMI, Stanford University School of Medicine, Stanford, CA 94305; ^c^Department of Structural Biology, Stanford University School of Medicine, Stanford, CA 94305

**Keywords:** invariant chain, HLA-DR, HLA-DQ, cryo-EM, antigen presenting

## Abstract

The loading of processed peptides on to major histocompatibility complex II (MHC-II) molecules for recognition by T cells is vital to cell-mediated adaptive immunity. As part of this process, MHC-II associates with the invariant chain (Ii) during biosynthesis in the endoplasmic reticulum to prevent premature peptide loading and to serve as a scaffold for subsequent proteolytic processing into MHC-II-CLIP. Cryo–electron microscopy structures of full-length Human Leukocyte Antigen-DR (HLA-DR) and HLA-DQ complexes associated with Ii, resolved at 3.0 to 3.1 Å, elucidate the trimeric assembly of the HLA/Ii complex and define atomic-level interactions between HLA, Ii transmembrane domains, loop domains, and class II-associated invariant chain peptides (CLIP). Together with previous structures of MHC-II peptide loading intermediates DO and DM, our findings complete the structural path governing class II antigen presentation.

## Significance

The structures of the full-length Human Leukocyte Antigen (HLA)-DR/DQ and invariant chain complexes provide insights into antigen presentation, revealing trimeric assembly details and the protective role of Ii in preventing premature peptide loading onto major histocompatibility complex II (MHC-II). Our findings demonstrate that invariant chain not only interacts with MHC-II via class II-associated invariant chain peptide (CLIP) peptide but also engages in polar interactions and transmembrane domain interactions. This work contributes to our understanding of antigen processing, laying a foundational structure for potential therapeutic exploration in MHC-II-related conditions.

The major histocompatibility complex class II (MHC-II) molecules play a crucial role against pathogens and foreign antigens in adaptive immune responses by presenting peptide antigens to CD4+ T lymphocytes (1, 2). The human MHC-II, or Human Leukocyte Antigen (HLA), consists of three isotypic loci—DP, DQ, and DR. These loci encode α and β subunits, forming heterodimeric MHC-II receptors, which enables the presentation of a diverse array of antigenic peptides (3).

**Fig. 1.**
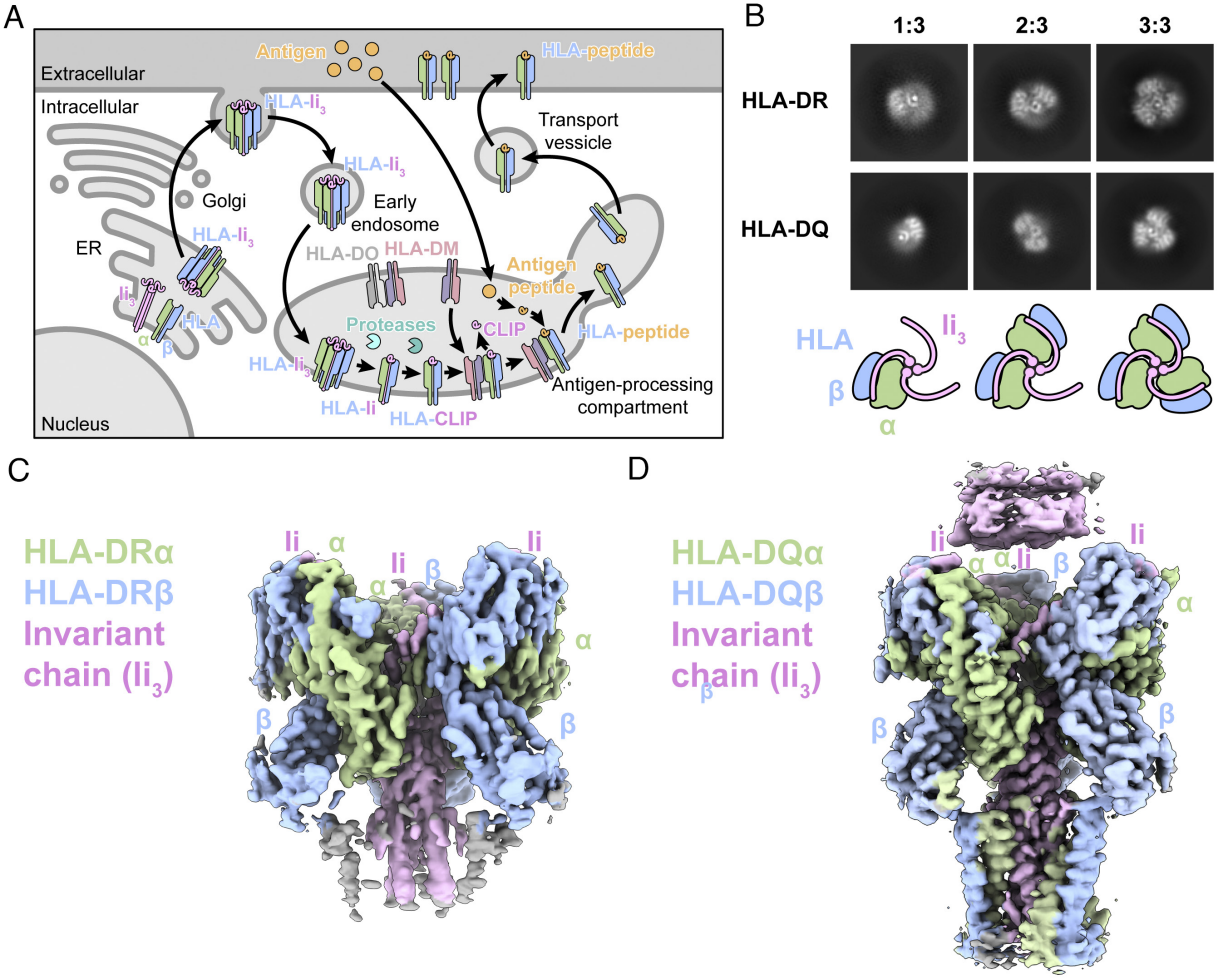
Cryo-EM maps and overall structures of HLA-DR/Ii and HLA-DQ/Ii complex. (*A*) Overview of MHC class II antigen presentation: MHC class II αβ dimers associate with invariant chain in the ER, forming 3:3:3 trimers. The complex traffics to endosomes, where invariant chain degrades by protease to CLIP, occupying the binding groove. HLA-DM facilitates CLIP removal, enabling peptide loading. HLA-DO can block HLA-DM. Once loaded with peptide, MHC class II molecules traffic to the plasma membrane. (*B*) Different oligomeric assembly states were observed for HLAαβ/Ii complex. Upper panel: representative 2D classification average of HLA-DR/Ii and HLA-DQ/Ii complex. Lower panel: representative cartoons of HLAαβ/Ii that correspond to Ii_3_:(αβ)_1_ (one MHC-II heterodimer per Ii_3_), Ii_3_:(αβ)_2_ (two MHC-II heterodimers per Ii_3_) and Ii_3_:(αβ)_3_ (three MHC-II heterodimers per Ii_3_) assembly state. (*C*) The density map of the HLA-DR/Ii trimer resolved is viewed from the side of the membrane. (*D*) The density map of the HLA-DQ/Ii trimer resolved is viewed from the side of the membrane. HLA-DRα/ HLA-DQα (pale green); HLA-DRβ/ HLA-DQβ (light blue); Ii (light pink).

The assembly and trafficking of MHC-II molecules are regulated processes involving invariant chain (Ii), which acts as a chaperone associated with MHC-II subunits during MHC-II biosynthesis and antigen processing (Fig. 1*A*). This association aids in the proper folding and serves to prevent premature binding of endogenous or exogenous ligands to MHC-II molecules, thereby ensuring that only antigenic peptides derived from extracellular sources will be presented (4, 5).

The Invariant chain, also known as CD74, is a single-spanning transmembrane (TM) protein exhibiting a type II topology. Consequently, its N terminus is oriented toward the cytosol, while the C-terminus is directed extracellularly. Previous studies have shown that Ii forms a homotrimer (Ii3), associating with MHC-II heterodimers to create a trimer of trimeric complex, Ii_3_:(αβ)_3_ (6, 7). This assembly is crucial for intracellular transport to endosomes, where Ii undergoes degradation by lysosomal proteases, giving rise to the class II-associated invariant chain peptide (CLIP) (8). CLIP lodges within the peptide-binding groove of MHC-II, acting as a “placeholder” to prevent premature peptide loading (9–11).

The structure of the MHC-II/Ii complex has been elusive and represents a gap in our structural understanding of MHC-II antigen processing. Several structures of several MHC-II–CLIP complex have been elucidated (12–19). While previous studies have offered insights into CLIP interactions with MHC-II molecules, a comprehensive understanding of the full-length complex has remained elusive. In this study, we resolved cryogenic electron microscopy (cryo-EM) structures of HLA-DR and HLA-DQ complexes bound to Ii, providing pivotal insights into this supermolecular chaperone assembly. The structural analysis of the invariant chain offers detailed insights into its trimerization interface, emphasizing conserved residues crucial for stability. Additionally, we elucidate atomic-level details of the binding interactions between HLA and the invariant chain, as well as CLIP.

## Results

### Structure of HLA–Ii Complexes.

We expressed protein by the BacMam method using human His-tagged HLA-DR15 (DRA*01:02/DRB1*15:01) and HLA-DQ (DQA1*05:01/DQB1*05:03) along with Flag-tagged Ii isoform p43. HEK293F cells were coinfected for optimal expression (20). Subsequently, we employed tandem affinity purification and size exclusion chromatography for protein complex purification in detergent (*SI Appendix*, Figs. S1 and S2). Cryo-EM images revealed well-dispersed particles, indicating homogeneous protein behavior. Results from two-dimensional classification distinctly demonstrated varying oligomeric assemblies of the HLAαβ/Ii complex. Specifically, 2D class averages of both HLA-DR/Ii and HLA-DQ/Ii complexes unveiled assemblies corresponding to three stoichiometries: Ii_3_:(αβ)_1_, Ii_3_:(αβ)_2_ asymmetric, and Ii_3_:(αβ)_3_ trimeric symmetric configurations (Fig. 1*B*). Physiologically, all three assemblies—3:1, 3:2, and 3:3—are known to coexist, with the 3:2 being particularly sensitive to proteolytic degradation within endosomes. In the case of the 3:3 trimer, the protease-resistant MHC-II heterodimers act as a shield, safeguarding Ii from degradation within endocytic compartments (21).

**Fig. 2.**
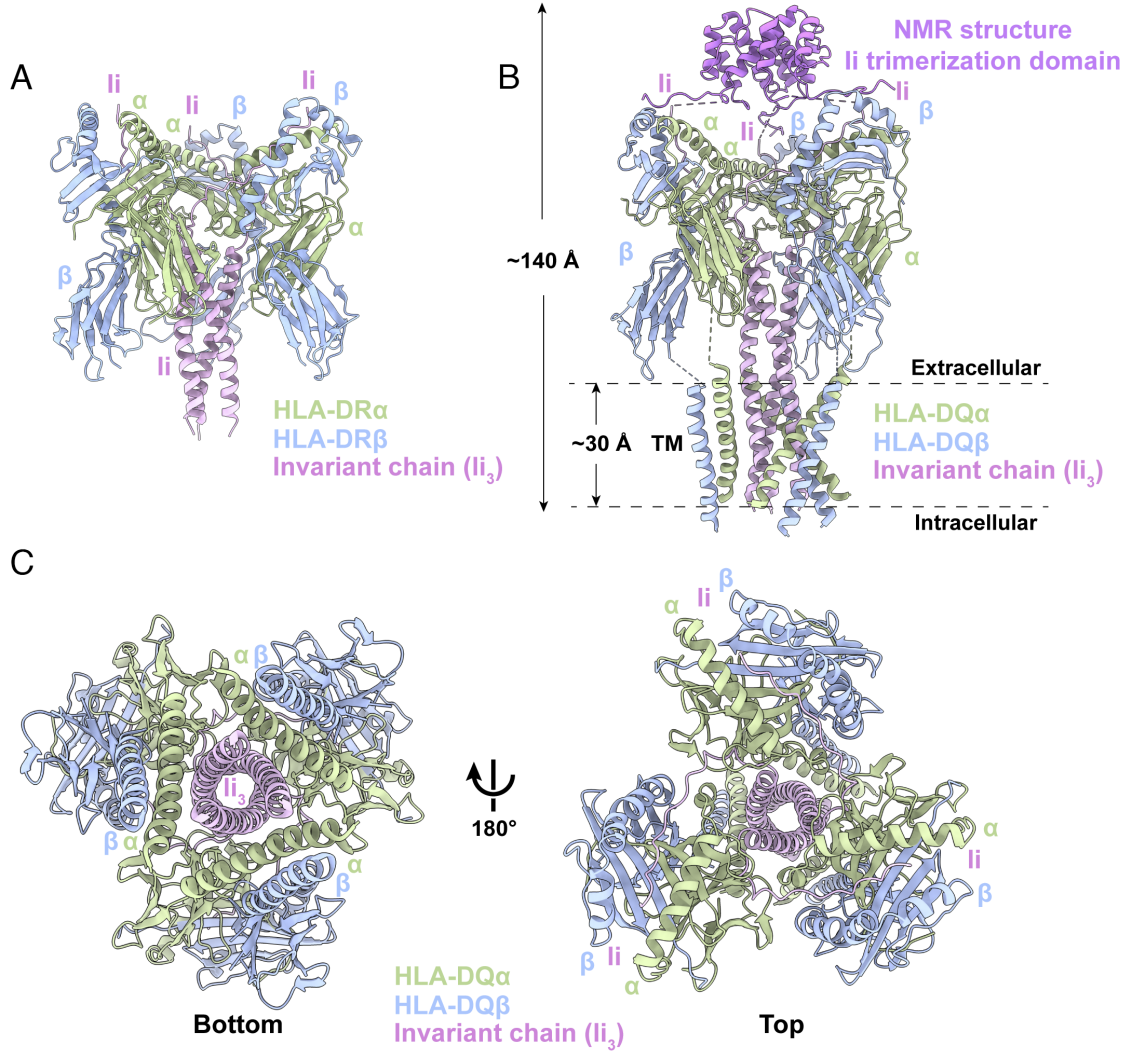
Atomic model of HLA-DR/Ii and HLA-DQ/Ii complex. (*A*) Ribbon diagram of the 3:3:3 HLA-DR/Ii complex. HLA-DRα/HLA-DQα (pale green); HLA-DRβ/HLA-DQβ (light blue); Ii (light pink). (*B*) Ribbon diagram of the 3:3:3 HLA-DQ/Ii complex. Ii invariant chain trimerization domain is docking with NMR structure (PDB:1IIE) colored as purple. (*C*) *Bottom* and *Top* views of the HLA-DQ/Ii complex.

We determined the structures of HLA-DR/Ii and HLA-DQ/Ii trimers to a final resolution of 3.0 and 3.1 Å, respectively (Fig. 1 *C* and *D* and *SI Appendix*, Figs. S3 and S4). Despite having similar resolution, the density map of HLA-DQ exhibits superior clarity. Within this map, the TM domain of both HLA-DQ alpha and beta chains is distinctly discernible. In contrast, the TM domain of HLA-DR is not resolved to the same extent. In the HLA-DQ/Ii map, although density is observable, a poly-A helix was constructed within the TM density for both HLA-DQα and HLA-DQβ (Fig. 2). The density of the trimerization domain, which situated C-terminal to CLIP within Ii (*SI Appendix*, Fig. S5), can be observed in 2D class averages and the initial 3D model (*SI Appendix*, Figs. S2 and S4). However, due to the inherent flexibility of this domain, the resolution was comparatively lower. To address this, we docked the previously determined NMR–derived structure of the Ii trimerization domain into our density maps for analysis (22).

The overall HLAαβ/Ii structures are approximately 140 Å in height and 80 Å in the longest dimension of width (Fig. 2*B*). These assembled complexes adopt a C3 symmetric trimeric configuration. In its top and bottom views (Fig. 2 *C* and *D*), the Ii component forms a central trimer through its C-terminal membrane-distal (the “trimerization domain”), and N-terminal TM and membrane proximal regions, flanked by the attachment of HLA-α and HLA-β. The HLA dimer engages with Ii through both extracellular and, unexpectedly, their TM domains to form a 9-stranded helical barrel within the plane of the membrane. The TM region of HLA-alpha (HLA-α) interacts with the TM domain of Ii, while concurrently, the HLA-beta (HLA-β) chain links with the TM domain of HLA-α (Fig. 2). This spatial arrangement weaves together the HLAαβ/Ii complex.

### Trimerization of the Ii Chain.

**Fig. 3.**
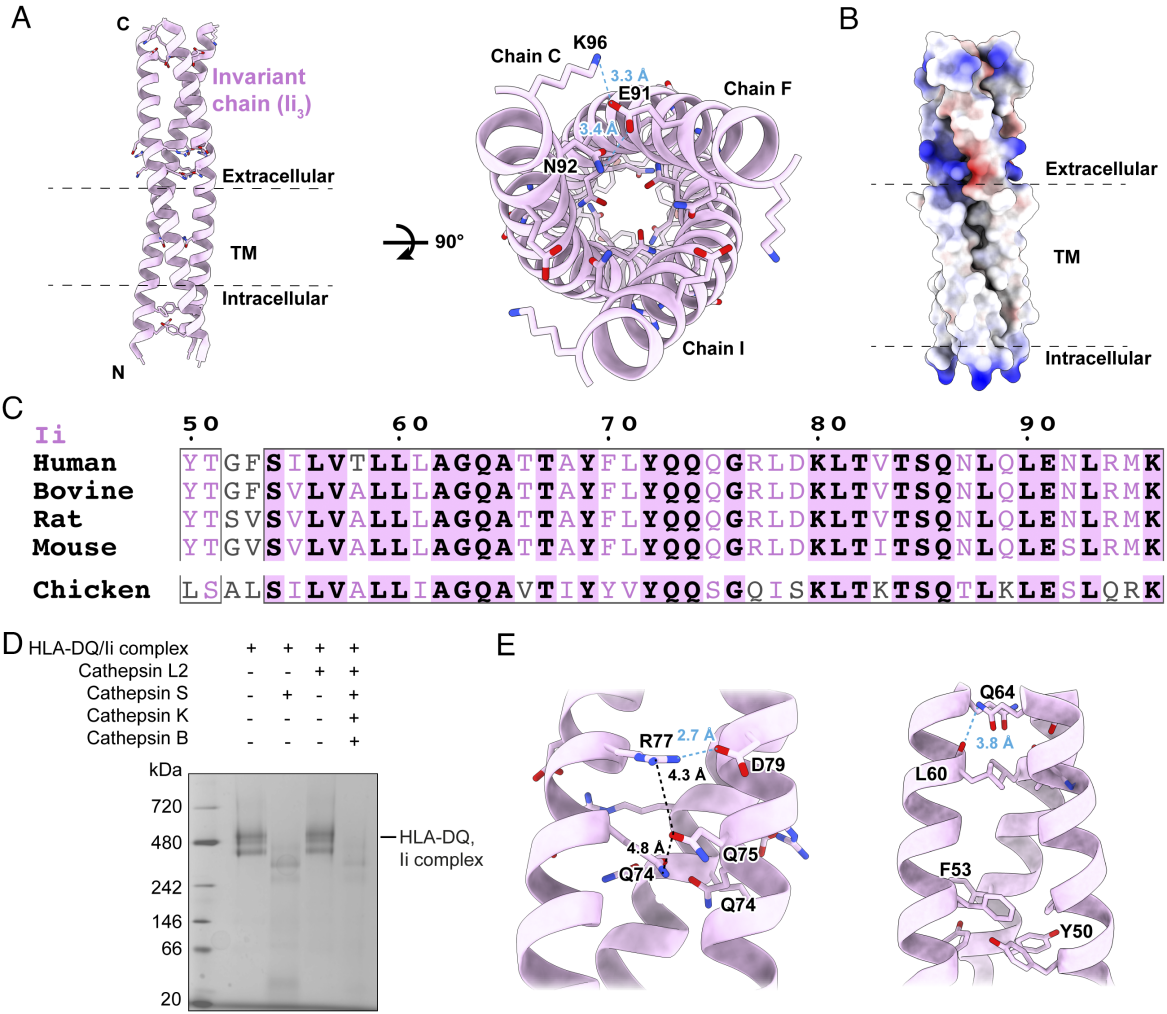
Structural analysis of invariant chain trimerization. (*A*) Binding residues between invariant chain. The residues that constitute the interface are shown as sticks in the two *Insets*. (*B*) TM Interface between invariant chain. The electrostatic potential was shown that the interface features many polar contacts. (*C*) Sequence alignment of invariant chain from different species. Invariant residues are bold and shaded with purple. Highly conserved residues are colored purple. (*D*) In vitro protease digestion assay. Purified recombinant HLA-DQ/Ii complex incubated with purified Cathepsins. Resulting fragments analyzed by Blue-native PAGE (BN-PAGE). (*E*) Subsequent panels show key residues for interaction on *Middle* (*Left*) and *Bottom* (*Right*) parts of the invariant chain helix. The cyan dashed lines represent hydrogen bond, and the black dashed lines indicate the distance between two atoms.

The assembly of Ii into its trimeric form is the initial step in the endosomal pathway. This trimerization process is reportedly mediated by the C-terminal luminal “trimerization domain” encompassing residues 134 to 208, whose NMR structure has been resolved (*SI Appendix*, Fig. S5) (22). In addition to the extracellular trimerization domains, our analysis unveils protein–protein interactions that play a role in stabilizing the trimeric structure of Ii, encompassing both the extracellular helix and the TM helix (Fig. 3*A*). The extracellular helix is stabilized by a hydrogen bonding network (Fig. 3*B*). Sequence alignment indicates that these polar residues are highly conserved across species (Fig. 3*C*).

We confirmed our structural analysis based on in vitro protease digestion assay. The full-length HLA-DQ/Ii trimer complex, with an estimated molecular weight of 480 kD, transformed into a stable band of around 350 kD upon Cathepsin S treatment rather than Cathepsin L2 treatment (Fig. 3*D*). Based on molecular weight considerations, this suggests that Cathepsin S likely targets the region of Ii between CLIP and the trimerization domain, leaving the oligomerization of the HLA-DQ/Ii trimer complex largely unaffected. This assay corroborates our structural analysis, affirming that beyond trimerization domains, other crucial protein–protein interactions also contribute to stabilizing the trimeric structure of Ii.

Residues proximal to CLIP, namely Lys96 and Glu91, form a charge-driven hydrogen bond network centered around Asn92 forming a hydrogen bond with Glu91. Additionally, near the membrane interface, Asp79 engages in a hydrogen bond network with Lys77, which in turn forms polar interactions with Gln75, and Gln75 reciprocates with Glu74, collectively establishing a network of stabilizing interactions (Fig. 3 *A* and *E*).

Prior investigations have emphasized the pivotal role of the TM domain in Ii trimerization, with particular emphasis on the significance of residue Gln64 (23). Mutations within the TM domain of Ii have been demonstrated to disrupt antigen presentation, primarily by destabilizing the Ii trimer. Our structural findings are consistent with prior results, demonstrating that Gln64 is capable of engaging in a polar interaction with carbonyl oxygen of Leu60 across different TM segments. We further identified aromatic residues Tyr50 and Phe53, which engage in a π–π interaction, thus contributing to the structural integrity of the trimeric assembly (Fig. 3*E*).

### Interaction of the Ii Chain with HLA Class II Molecules.

**Fig. 4.**
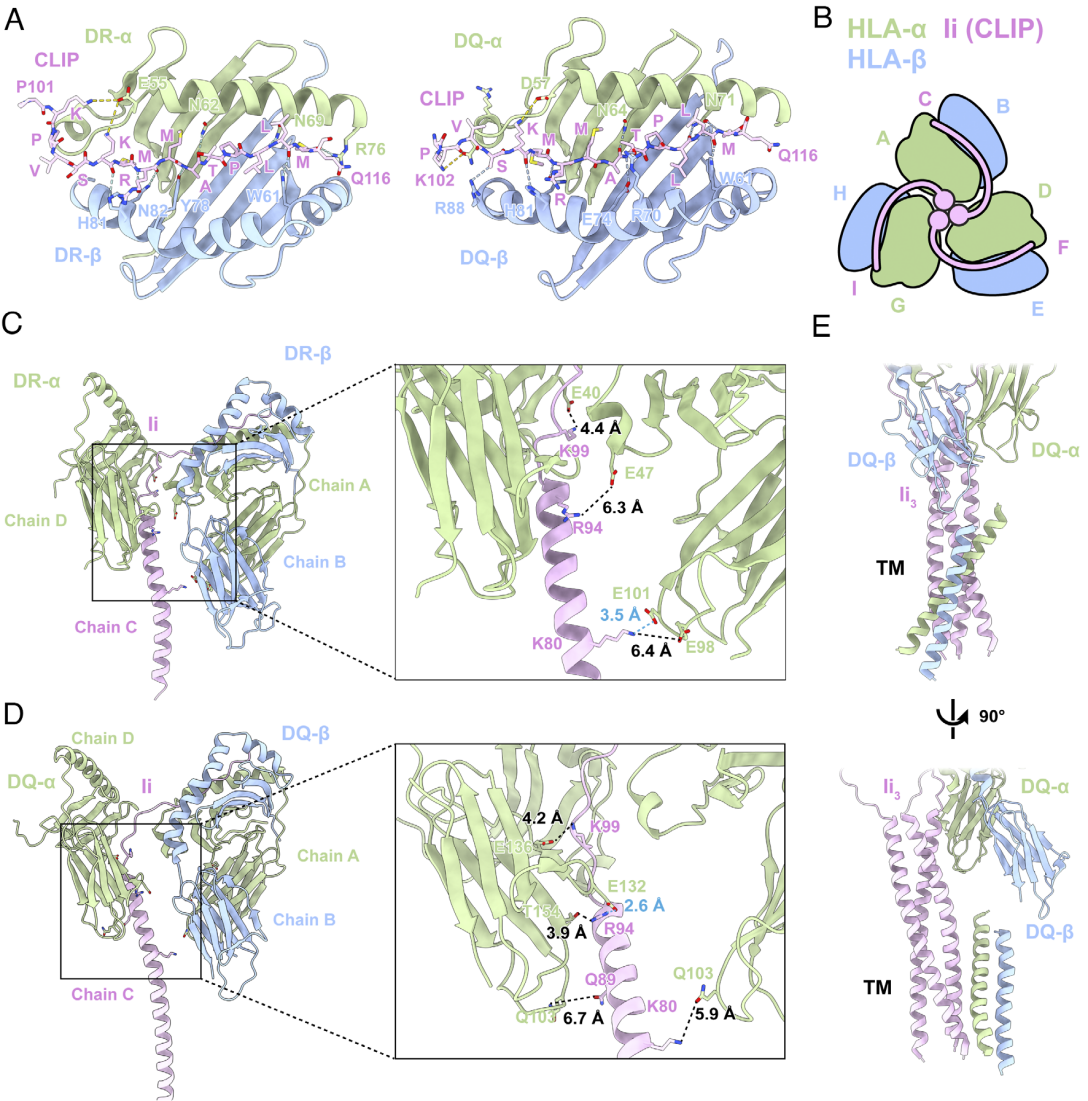
Binding between HLA and invariant chain. (*A*) *Left*: The interaction details between HLA-DR and CLIP. *Right*: The interaction details between HLA-DQ and CLIP. CLIP is shown as backbone and side chains. HLA-DR is shown as cartoon. Hydrogen bond between CLIP backbone and HLA side chain is shown as blue dashed lines. Interaction between CLIP side chain and HLA side chain is shown as yellow dashed lines. (*B*) Chain IDs of the HLAαβ/Ii 3:3:3 trimer complex. (*C*) The interaction details between HLA-DR and Ii TM plus loop domain. Key binding residues are shown as sticks. The cyan dashed lines represent hydrogen bond, and the black dashed lines indicate the distance between two atoms. (*D*) The interaction details between HLA-DQ and Ii TM plus loop domain. Key binding residues are shown as sticks. (*E*) The interaction between HLA-DQ TM and Ii TM. A-DQ TM and Ii TM.

HLA-DR and HLA-DQ bind to the CLIP region in a way that closely mirrors findings from previous studies (Fig. 4*A* and *SI Appendix*, Fig. S6 *A* and *B*) (12, 18). Within this interaction, hydrogen bonds predominantly form between the main chain of CLIP and the side chains of HLA-α and HLA-β governing this molecular association. Polar interactions also occur between the CLIP side chains and specific residues on HLA-α. Specifically, in our study, Lys102 and Lys106 on CLIP exhibit the potential for forming polar interactions with Glu55 in HLA-DRα, as well as interactions with Gln53 and Asp57 on HLA-DQ (Fig. 4*A*).

Moving beyond the central interaction involving CLIP and the groove of HLA Class II, the extracellular loop and helix of Ii engage with HLA-α. For clarity, we have labeled the chain IDs of the trimeric 3:3:3 complex, emphasizing the specific contributions of each chain to the molecular interactions (Fig. 4*B*). A single Ii molecule binds to two HLA molecules by means of a strand extension mediated by a linker region between the CLIP peptide and the loop region. The interaction between Ii (chain C) and its own HLA-DRα (chain A) extends beyond the previously observed engagement with CLIP and the groove. This expanded interaction involves a hydrogen bond between Lys80 on Ii and Glu101 on HLA-DRα (Fig. 4*C*). Glu132 on the adjacent HLA-DQα (chain D) engage in a hydrogen bond with Arg94 on Ii (chain C) (Fig. 4*D*). These multisite interaction points resolved by our structure present potential targetable sites for therapeutic strategies aimed at modulating MHC-II antigen presentation.

While we only modeled a poly-A helix into the TM density for HLA-DQα and HLA-DQβ, the TM domains of HLA-DRα, HLA-DRβ, HLA-DQα, and HLA-DQβ reveal a prevalence of hydrophobic residues (*SI Appendix*, Fig. S6). In addition to polar interactions within the extracellular domain, the TM region of HLA-α interacts with the TM domain of Ii, primarily through hydrophobic interactions within the confined contact area (Fig. 4*E*). Simultaneously, the HLA-β chain interacts with the TM region of HLA-α.

## Discussion

**Fig. 5.**
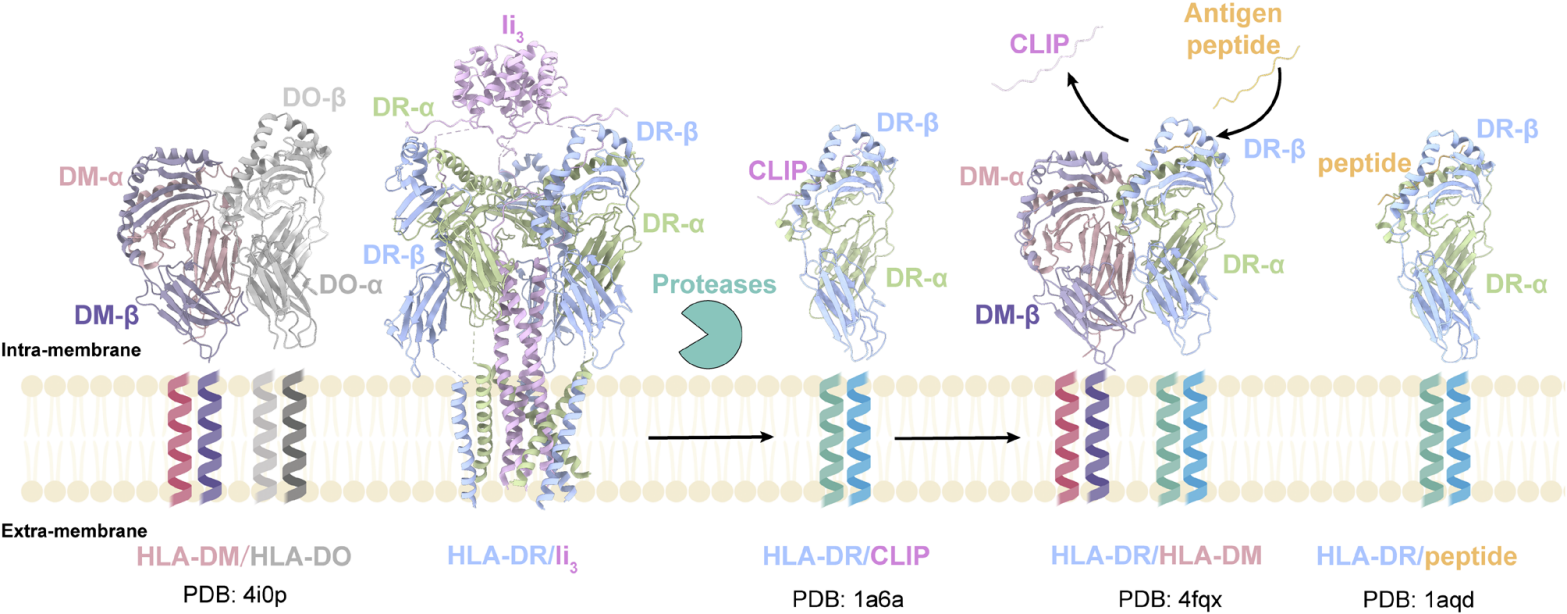
Structural overview of MHC class II antigen presentation cycle. These structures collectively illustrate key steps in antigen presentation, from the initial assembly to peptide loading. HLA-DM/HLA-DO structure (PDB:4iop). HLA-DO interacts with and inhibits HLA-DM. HLA-DR/Ii structure. MHC class II αβ dimers associate with the invariant chain (Ii), forming 3:3:3 trimers. HLA-DR/CLIP structure (PDB:1a6a). The HLA-DR/Ii complex degrades by protease, leading to CLIP occupancy in the binding groove. HLA-DR/HLA-DM structure (PDB:4fqx). HLA-DM facilitates CLIP removal from HLA-DR, enabling peptide loading. HLA-DR/peptide structure (PDB:1aqd). Upon peptide loading, MHC class II molecules traffic to the plasma membrane.

The role of Ii in MHC-II antigen presentation has been well studied (24–28). Our cryo-EM structures of the HLA-DR/Ii and HLA-DQ/Ii complexes, resolved at 3.0 to 3.1 Å, offer detailed insights into the full-length complex, rationalizing a wealth of structure–function data (29–31), and together with prior structures of other intermediates containing DM and DO (12, 32–34), complete the final missing piece of the puzzle of structures in MHC-II antigen presentation cycle (Fig. 5). While these structures, until now, have largely been useful for rationalizing structure–function data, these complexes also present possible therapeutic targets for modulating immunity, which has been a largely underexplored strategy.

The trimerization of Ii has been previously reported and is known to be mediated by its trimerization domain and TM domain (35, 36). Our structural analysis emphasizes the critical polar interactions stabilizing the trimeric structure of Ii, encompassing both extracellular helix and TM helix binding. Key hydrogen bonds, including Lys96–E91, Asn92–E91, and Arg77–Asp79, play a crucial role in the Ii trimerization process. Notably, even after digestion of the trimerization domain by Cathepsin S, the HLA-DQ/Ii complex remains capable of forming a trimer, affirming the crucial protein–protein interactions between Ii helix.

The HLA/Ii complex architecture unveils detailed interaction between Ii and HLA αβ. Notably, beyond the CLIP–HLAαβ groove interaction, our study highlights the extracellular loop and helix of Ii with HLA-α, contributing to complex stability. This involvement is underscored by the formation of hydrogen bonds, specifically between Lys80 on Ii and Glu101 on HLA-DR, as well as between Arg94 on Ii and Glu132 on HLA-DQ chain D. Our study also indicates the TM interaction exits between Ii TM and HLA-α TM, while the HLA-beta chain interacts with the HLA-alpha TM domain. While CLIP binding dominates in energy contribution, other interactions with Ii are delicate and can easily dissociate postcleavage, making this configuration apt for antigen surface representation.

In conclusion, our study advances the understanding of the HLA/Ii complex, offering a structural framework that enriches our comprehension of antigen presentation mechanisms. Future studies exploring isoform-specific effects and sequential Ii processing steps could deepen our comprehension of this pivotal immune response mechanism.

## Materials and Methods

### Protein Expression and Purification.

The cDNAs of full-length human full-length invariant chain (Uniprot ID: P04233), HLA-DRA*01:02 (Uniprot ID: P01903), HLA-DRB1*15:01 (Uniprot ID: P01911), HLA-DQA1*05:01 (Uniprot ID: P01909), and HLA-DQB1*05:03 (Uniprot ID: P01920) were cloned individually into pVLAD6 vector (Addgene plasmid # 41850). Ii was fused with a N-terminal Flag-tag, while HLA-DRA, HLA-DRB, HLA-DQA, and HLA-DQB were fused with a C-terminal His-tag. Ii/HLA-DRA/HLA-DRB complex and Ii/HLA-DQA/HLA-DQB complex were coexpressed using the BacMam system in HEK293F cells (20).

The protein was solubilized from 293F cell membranes with 2% (w/v) n-dodecyl-β-D-maltoside (DDM, Anatrace) and 0.2% (w/v) cholesteryl hemisuccinate tris salt (CHS, Anatrace) at 4 °C for 1.5 h and purified using nickel affinity resin (Ni-NTA, Qiagen). The protein was washed with the buffer containing 25 mM Tris pH 8.0, 150 mM NaCl, 20 mM imidazole, 0.02% (w/v) DDM, and 0.0002% (w/v) CHS then eluted with the wash buffer plus 200 mM imidazole. The eluent was then applied to Flag-M2 resin (Sigma). After being washed with the buffer containing 25 mM Tris pH 8.0, 150 mM NaCl, 0.02% (w/v) DDM, and 0.0002% (w/v) CHS, the protein was eluted by wash buffer plus 0.2 mg/mL Flag peptide (CST). The protein was then concentrated and subjected to size-exclusion chromatography (Superdex-200, Cytiva) that was pre-equilibrated in the buffer containing 25 mM Tris pH 8.0, 150 mM NaCl, and 0.005% (w/v) lauryl maltose neopentyl glycol (LMNG, Anatrace). Peak fractions were collected and concentrated to 6 mg/mL for cryo-EM analysis.

### Data Collection and Structure Determination.

To prepare the cryo-EM specimens, 3.0 μL of protein complex was applied to a glow-discharged grid (Quantifoil Au R1.2/1.3, 300 mesh) and excess sample was blotted with a filter paper for 3.0 s before plunge-freezing in liquid ethane cooled by liquid nitrogen with Vitrobot (Mark IV, Thermo Fisher Scientific) at 8 °C and 100% humidity. Cryo-EM movies were collected using an FEI Titan Krios operated at 300 kV equipped with Gatan K3 camera in counting mode. Micrographs of the HLA-DR/Ii and HLA-DQ/Ii complex were recorded using SerialEM (37) in the super-resolution mode with a pixel size of 0.653 Å and 0.4195 Å, respectively. Each stack of 50 frames was exposed for 4.5 s with 0.09 s exposure per frame at an exposure rate of ~16 e^−^/s/Å^2^ at the specimen, and the defocus range between −1.0 and −2.0 μm. Collected movies were processed and assessed with cryoSPARC Live. Patch motion correction was performed with binning to the physical pixel sizes of 1.306 and 0.839 Å, respectively.

For the HLA-DR/Ii structure, curated 11,059 movies were used for the downstream processing with cryoSPARC (38). Then, 9,929,368 particles are extracted from micrographs with the 448/122 binned box. Two rounds of 2D classifications were used to remove contaminants, yielding 825,755 particles that appeared to be protein. In addition, 1-class ab initio reconstruction followed by an initial nonuniform (NU) refinement generated a 5.5 Å map. After re-extracting particles with the 448/224 binned box and followed by two rounds of heterogeneous refinements generated 598,412 particles set for further NU-refinement. The best model and set of particles were then subjected to several rounds of local refinement, local contrast transfer function (CTF) refinement, and global CTF refinement, resulting in a 3.03 Å map.

For the HLA-DQ/Ii structure, 14,450 curated movies were used for the downstream processing with cryoSPARC and 7,843,242 particles were extracted from micrographs with the 560/140 binned box. After two rounds of 2D classifications, Then, 895,894 particles were selected followed by NU-refinement generating a 3.5 Å map. For further improving the density, seed-facilitated guided multireference 3D classification was performed (39). The multireferences include an accurate reference and three biased references. After removing duplicated particles and re-extracting particles with the 560/280 binned box, a 3.5 Å map was generated with 891,429 particles following by NU-refinement. 3D classification with resolution gradient maps (3.5 Å map and two other maps with low-pass filtered resolution of 8 Å and 16 Å), and noise reweighted maps (3.5 Å map and two other maps that micelle was downscaled by a factor of 0.3 and 0.7) further improved resolution. 421,582 particles were selected and then subjected to global CTF refinement, local CTF refinement, and local refinement, resulting in a 3.12 Å map.

The resolution was estimated with the gold-standard Fourier shell correlation 0.143 criterion. The angular distributions of the particles used for the final reconstruction of the HLA-DR/Ii and HLA-DQ/Ii complex are sufficient to produce maps with low levels of anisotropy.

### Model Building and Refinement.

The atomic coordinates of the HLA-DR/Ii and HLA-DQ/Ii complexes were generated by combining homology modeling and de novo model building. The atomic coordinates of the Ii were initially generated by AlphaFold models (40, 41). The structure was then docked into the density map using UCSF Chimera X (42) and manually adjusted and rebuilt by COOT (43). Crystal structures of the extracellular domain of HLA-DR (PDB code: 3PDO) and HLA-DQ (PDB code: 5KSU) were used and fitted into the density, individually (14, 18).

The models of HLA-DR/Ii and HLA-DQ/Ii complexes were refined against the corresponding map using PHENIX in real space with secondary structure and geometry restraints (44). The structures of the HLA-DR/Ii and HLA-DQ/Ii complexes were validated through examination of the Molprobity scores and statistics of the Ramachandran plots (*SI Appendix*, Table S1). Molprobity scores were calculated as described (45).

### Protease Digestion Assay.

First, 20 ng purified protease (Cathepsin S, Cathepsin L2, Cathepsin K, Cathepsin B) was preactivated in 10 μL activation buffer (400 mM sodium acetate, 4 mM EDTA, 8 mM DTT in pH 5.5) on ice for 15 min. Then, 4 μg protein complex was added and incubated with protease at 37 °C for 1 h. Proteins were analyzed by Blue-Native PAGE.

## Supplementary Material

Appendix 01 (PDF)

## Data Availability

Cryo-EM maps and atomic coordinates for the HLA-DR/Ii and HLA-DQ/Ii structures have been deposited in the PDB (http://www.wwpdb.org) with the accession codes 8VRW (46), 8VSP (47), and in the Electron Microscopy Data Bank (https://www.ebi.ac.uk/emdb/) with the accession codes EMD-43488 (48), EMD-43501 (49), respectively. All study data are included in the article and/or *SI Appendix*.
