# Supplementary material for: Structural insights into human MHC-II association with invariant chain
Source: Proc Natl Acad Sci U S A. 2024 Apr 30;121(19):e2403031121. doi: 10.1073/pnas.2403031121 (PMC11087810; doi:10.1073/pnas.2403031121)
Supplement: Supplementary file 1 — Appendix 01 (PDF) [file pnas.2403031121.sapp.pdf]

Supporting Information for
Structural Insights into human MHC-II association with Invariant Chain

Nan Wang, Deepa Waghray, Nathanael A. Caveney, Kevin M. Jude, and K. Christopher Garcia

K. Christopher Garcia
Email: kcgarcia@stanford.edu

This PDF file includes:

Figures S1 to S6
Tables S1

Fig. S1. Characterization of the human HLA-DR/Ii complex.

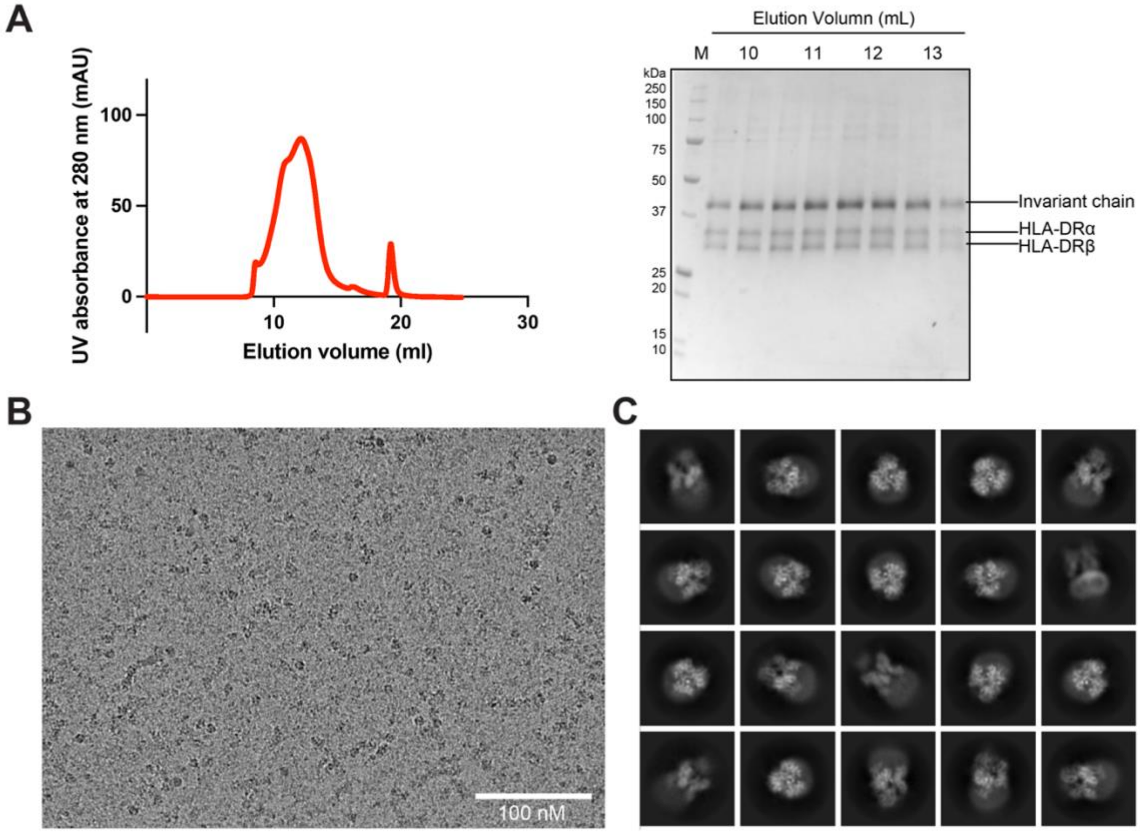

(A) Size exclusion chromatography of HLA-DR/Ii complex after two-step affinity purification. Shown on the right is SDS-PAGE visualized by Coomassie blue staining. Three bands labeled indicates the invariant chain, HLA-DRα1, and HLA-DRβ1 subunit. (B) A representative micrograph of the HLA-DR/Ii complex purified in LMNG. Scale bar = 100 nm. (C) Representative 2D class averages of the HLA-DR/Ii complex.

Fig. S2. Characterization of the human HLA-DQ/Ii complex.

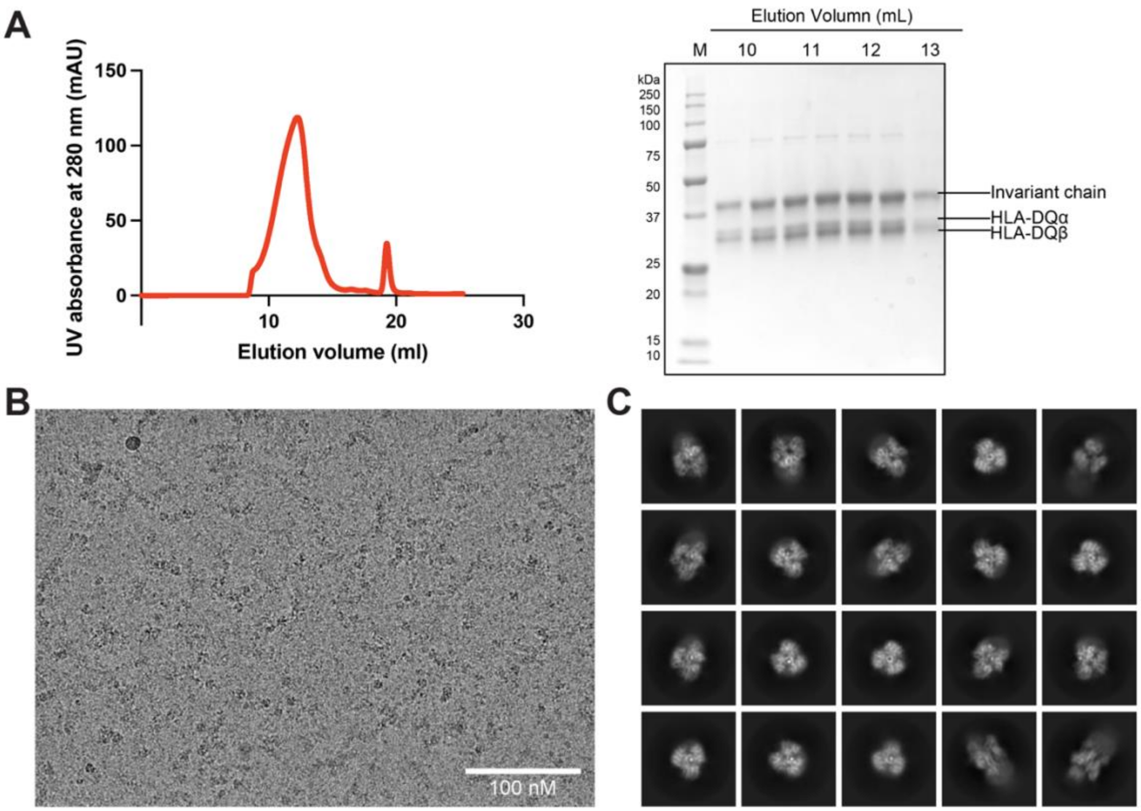

(A) Size exclusion chromatography of HLA-DQ/Ii complex after two-step affinity purification. Shown on the right is SDS-PAGE visualized by Coomassie blue staining. Three bands labeled indicates the invariant chain, HLA-DQα1, and subunit HLA-DQβ1. (B) A representative micrograph of the HLA-DQ/Ii complex purified in LMNG. Scale bar = 100 nm. (C) Representative 2D class averages of the HLA-DQ/Ii complex.

Fig. S3. Cryo-EM analysis of the human HLA-DR/Ii complex.

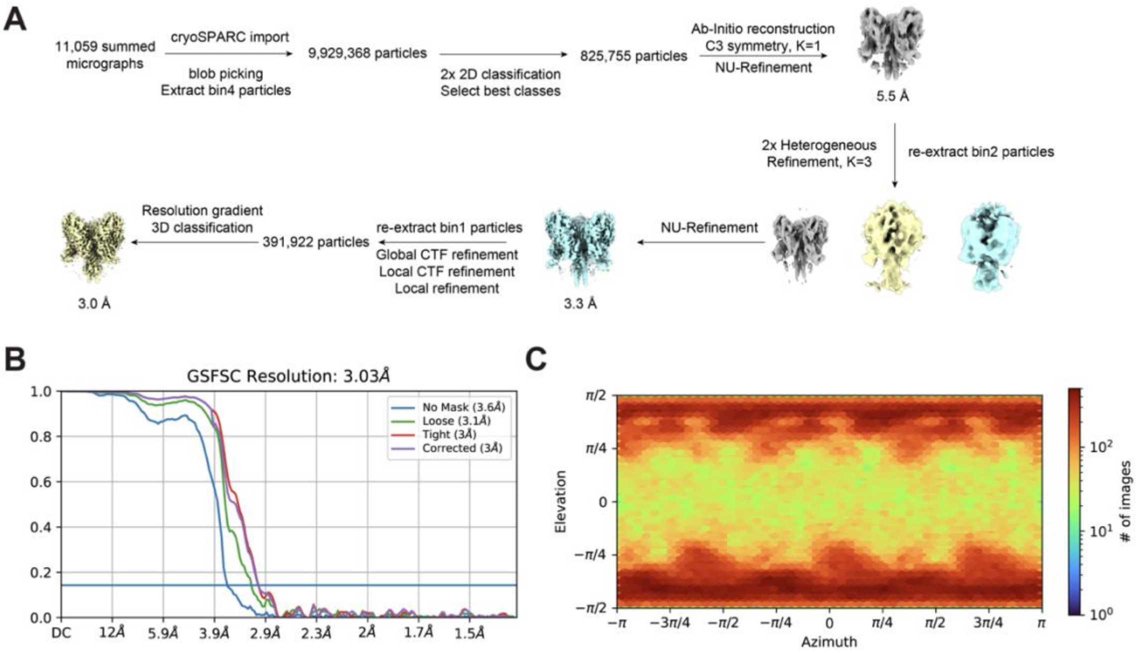

(A) Flow chart for data processing of the HLA-DR/Ii complex. Details are described in Material and Methods. (B) Gold standard Fourier shell correlation (FSC) curve for the 3D refinement of the overall structure of the HLA-DR/Ii complex calculated in CryoSPARC. (C) Angular distribution of the particles used for the final reconstructions.

Fig. S4. Cryo-EM analysis of the human HLA-DQ/Ii complex.

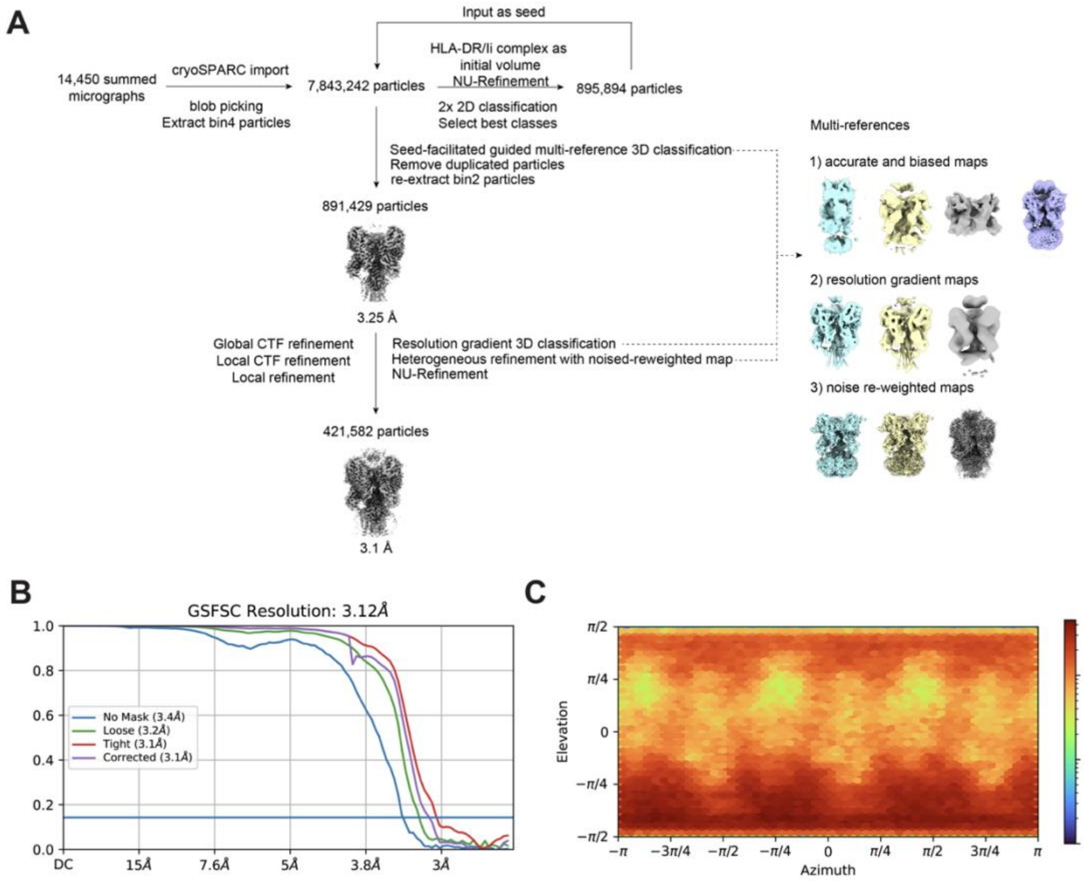

(A) Flow chart for data processing of the HLA-DQ/Ii complex. Details are described in Material and Methods. (B) Gold standard Fourier shell correlation (FSC) curve for the 3D refinement of the overall structure of the HLA-DQ/Ii complex calculated in CryoSPARC. (C) Angular distribution of the particles used for the final reconstructions.

Fig. S5. Primary structures of the human Ii isoforms.

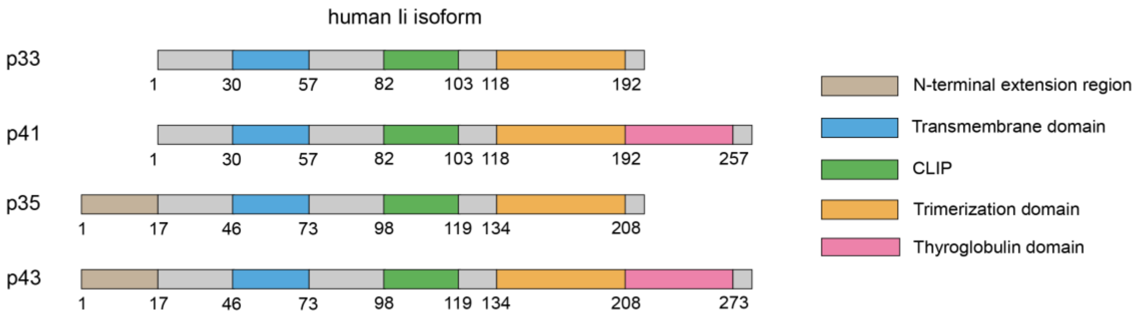

The primary structures of four different human invariant chain isoforms (p33, p41, p35 and p43). CLIP, the class II-associated invariant chain peptides.

Fig. S6. Densities for the representatives of the human HLA-DR/Ii complex and HLA-DQ/Ii complex.

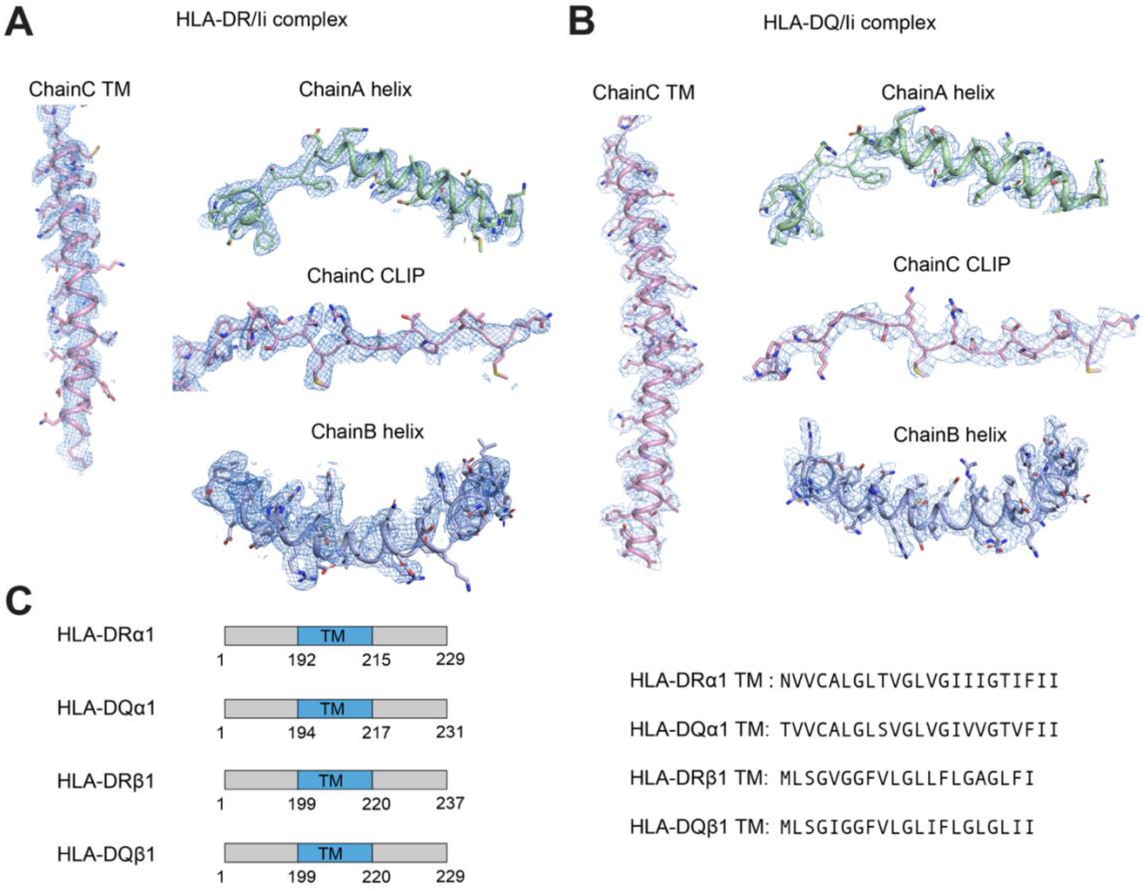

(A-B) Local densities for (A) HLA-DR/Ii and (B) HLA-DQ/Ii complex, shown as blue meshes, were prepared in PyMol at 3 σ contour level. (C) The primary structures and TM sequences of human HLA-DRα, HLA-DRβ, HLA-DQα, HLA-DQβ.

Table S1. Summary of data collection and model statistics.

	HLA-DR/Ii	HLA-DQ/Ii
**Data collection**		
EM equipment	FEI Titan Krios	
Voltage (kV)	300	
Detector	K3	
Pixel size (Å)	0.653	0.4195
Electron dose (e-/Å^2)	60	60
Defocus range (μm)	1.0~2.0	1.0~2.0
**Reconstruction**		
Software	CRYOSPARC	
Number of used Particles	391,922	421,582
Symmetry	C3	C3
Map sharpening Method	deepEMhancer (visualization) uniform B-factor of 200 Å^2	
Final Resolution (Å)	3.03	3.12
**Model building and refinement**		
Software	PHENIX & COOT	
Initial models used (PDB codes)	3PDO	5KSU
Model composition		
Non-hydrogen atoms	10413	11388
Protein residues	1272	1476
Ligand	——	NAG
B factors (Å)		
Protein	80.06	68.24
Ligand	——	120.22
R.m.s deviations		
Bonds length (Å)	0.005	0.005
Bonds Angle (˚)	0.956	0.944
Ramachandran plot statistics (%)		
Preferred	95.69	96.67
Allowed	4.31	3.33
Outlier	0.0	0.0
Validation		
Molprobity score	2.07	1.68
Clash score	18.92	8.71
Rotamer outliers (%)	0.52	0.51
